# Prevalence and patterns of abnormal metabolic screening in pediatric acute encephalopathy: a PICU study from Egypt

**DOI:** 10.1007/s00431-026-06939-x

**Published:** 2026-04-23

**Authors:** Marwa Ibrahem Abdelrazic, Mohamed S. Hemeda, Manar Anwar Abd-Elaziz, Ahmed Roshdy Mahmoud, Ibtehal Saad Abuelela

**Affiliations:** 1 Department of Paediatrics, Faculty of Medicine, Minia University, El-Minia, Egypt; 2 Department of Forensic Medicine and Clinical Toxicology, Port Said University, Port Said, Egypt

**Keywords:** Acute encephalopathy, Inborn errors of metabolism, Metabolic screening, Tandem mass spectrometry, GC–MS, Mortality predictors, Forensic Medicine, Sudden Unexplained Death, Postmortem Metabolic Screening

## Abstract

**Background:**

Inborn errors of metabolism (IEMs) remain underrecognized in children presenting with acute non-infectious encephalopathy, where timely diagnosis and treatment can be lifesaving. This study sought to estimate the yield and spectrum of metabolic screening patterns suggestive of IEM among children with presumed non-infectious acute encephalopathy admitted to a tertiary pediatric intensive care unit in Egypt.

**Methods:**

From March 2023 to June 2024, we undertook and finalized a cross-sectional research study at the Minia University PICU. After initial clinical evaluation, cerebrospinal fluid (CSF) analysis did not support overt central nervous system infection; children with presumed non-infectious acute encephalopathy were enrolled. They underwent standardized clinical, laboratory, and neuroimaging workups. Metabolic screening comprised tandem mass spectrometry (TMS) of amino acids/acylcarnitines and urine organic acids by gas chromatography–mass spectrometry (GC–MS). N = 100 participants were included.

**Results:**

Abnormal metabolic screening suggestive of an underlying IEM was identified in 26% (n = 26) of children with non-infectious encephalopathy (NIE). The most common abnormal profiles were amino acid disorders (38.5%), organic acidemias (34.6%), and fatty acid oxidation defects (26.9%). Overall mortality was 59%. Abnormal screening was associated with higher ammonia levels (median 176 vs 65 μmol/L; p < 0.001) and a higher frequency of cognitive delay, delayed motor development, and hypotonia (all p < 0.05). On ROC analysis, ammonia > 93.5 μmol/L predicted abnormal screening (sensitivity 84.6%, specificity 65%). In univariate logistic regression, higher serum ammonia, cognitive delay, and delayed motor development were associated with abnormal metabolic screening. In a parsimonious multivariable model, higher ammonia (adjusted OR 1.010, 95% CI 1.004–1.015; p = 0.001) and delayed motor development (adjusted OR 3.66, 95% CI 1.24–10.86; p = 0.019) remained independent predictors.

**Conclusions:**

Abnormal metabolic screening suggestive of IEMs comprises a substantial proportion of pediatric NIE in our setting. Early metabolic screening coupled with readily available biomarkers (notably ammonia) may help prioritize patients for confirmatory testing and timely management.

## Introduction

Inborn errors of metabolism (IEMs) are conditions that are sometimes neglected, despite their undeniable importance, even among the most critical pediatric patients [1]. While IEMs are complex and highly diverse, each disorder results from a deficiency in one or more critical regulatory enzymes in fundamental biochemical pathways, leading to the uncontrolled buildup of noxious metabolites and deficiencies in energy production [2]. The IEMs collectively exert impact across several organ systems, and when unsupported, can culminate in irreversible, debilitating neurodisability or death [3].

Although IEMs are individually uncommon, their combined importance is significant, given the global incidence of approximately 1 per 2000 live births (50/100,000) [4]. Patients with IEMs for whom early presymptomatic diagnosis is possible have markedly improved outcomes due to focused or neonatal metabolic screening [5].

Inborn errors of metabolism (IEMs) must be detected in critically ill children, even though they are often missed [1]. Avoiding an irreversible neurological decline is critical, so the need for premature diagnosis is reinforced by the cumbersome processes for managing such patients, primarily because of the attacks' nonspecific nature [6]. IEMs are unique and intricate genetic disorders in which biochemical pathways stall due to missing catalytic activities, leading to energy deficiency and the accumulation of toxic by-products [2, 7]. If IEMs are not treated, the outcomes can be disastrous, with irreversible neurological deficits and systemic body failure eventually resulting in death [3].

IEMs, in their untreated form, can be life-threatening, which leaves children with multiple avenues for severe disability. Most start during the first year of life but can also begin decades later, even in adulthood [3].

Tandem mass spectrometry (TMS) is fundamental in modern newborn screening. It can identify over 30 metabolic disorders in a single dried blood spot with remarkable sensitivity and specificity [8, 9]. These assays can detect disorders of amino acid metabolism, organic acidemias, defects in fatty acid oxidation, and urea cycle disorders.

In Egypt, the newborn screening program is limited to the detection of congenital hypothyroidism and phenylketonuria (PKU). However, information on the broader burden and the clinical spectrum of inborn errors of metabolism (IEMs) in children is scarce. These studies were often limited to specific disorders or geographic areas.

This study aimed to identify the biochemical features and clinical manifestations of inborn errors of metabolism (IEMs) in children presenting to the Pediatric Intensive Care Unit (PICU) with unexplained acute non-infectious encephalopathy (NIE). By improving early recognition of children at higher risk of an underlying IEM, such an approach may facilitate earlier targeted treatment, reduce preventable neurological injury, and ultimately improve longer-term neurodevelopmental outcomes.

From the viewpoint of forensic medicine, IEMs and associated metabolic encephalopathies may represent an essential but overlooked reason for unexplained coma or sudden death in infants and children. In these cases, forensic medicine may benefit from considering metabolic screening to differentiate natural from toxic or hypoxic causes.

## Methods

### Study design and setting

We conducted a cross-sectional study at the Pediatric Intensive Care Unit (PICU) and the Pediatric Medicine & Surgery Hospital, Faculty of Medicine, Minia University (Egypt), from March 2023 to June 2024. Written and verbal informed consent was obtained from parents or legal guardians. The Ethical Committee of Minia College of Medicine approved the study protocol in accordance with the Declaration of Helsinki (Approval No. 526/2022).

### Participants

Consecutive children presenting with acute encephalopathy were screened for eligibility. The study population comprised children with presumed non-infectious acute encephalopathy, as determined by initial clinical evaluation, and cerebrospinal fluid (CSF) analysis that did not support an overt central nervous system infection. Inclusion required a disturbed level of consciousness lasting ≥ 6 h (unresponsiveness, abnormal response to stimuli, loss of sleep–wake cycle, or absence of purposeful activity), together with one or more compatible acute features such as coma, lethargy, persistent convulsions, hypoglycemia, vomiting, or diarrhea. Children were enrolled when the treating team considered an inherited metabolic disorder or other non-infectious metabolic cause within the differential diagnosis after the initial assessment. Exclusion criteria were a history of chromosomal disorders, documented toxic exposure, brain tumors, confirmed central nervous system infection, perinatal brain injury, or comparable conditions.

### Clinical assessment

All enrolled children underwent a standardized evaluation that captured perinatal data, detailed family pedigree, and a comprehensive general and neurological examination, including anthropometric measurements and developmental assessment. Seizure semiology at presentation was pragmatically classified from the clinical record, where feasible, in alignment with the ILAE 2017 framework, as generalized-onset motor seizures, focal-onset seizures, myoclonic seizures, mixed/unclassified seizure presentations, or no seizures. This classification was based on the predominant documented clinical seizure pattern at presentation rather than on formal electroclinical epilepsy classification.

### Laboratory work-up

Baseline investigations included complete blood count, renal and liver function tests, serum electrolytes, random blood glucose, serum ammonia and lactate, and cerebrospinal fluid (CSF) analysis. Children were classified as having presumed non-infectious encephalopathy after the initial clinical evaluation, and CSF findings did not support overt central nervous system infection. Microbiological cultures and/or molecular testing (e.g., PCR) were not performed systematically for all enrolled cases; rather, these investigations were requested selectively according to the treatment team's clinical judgment, the level of suspicion for infection, and local test availability during acute PICU care. Because these investigations were not part of a uniform study-mandated protocol for all enrolled children, the exact number of children who underwent additional microbiological and/or molecular testing was not systematically captured in the study dataset. Toxic exposure was assessed based on caregiver history and clinical evaluation; routine toxicology/drug screening was not performed. Children with unexplained presumed non-infectious encephalopathy then underwent metabolic screening on admission.

### Metabolic screening (TMS and GC–MS)

Dried blood spots (Whatman 903) were prepared from venous or heel-prick samples. On a Waters Alliance 2790 HPLC equipped with a triple quadrupole, amino acids and acylcarnitines were quantified by MS/MS, using Phe/Tyr, Leu/Phe, C3/C2, and C3-DC/C10 ratios. Internal standards were from Cambridge Isotope Laboratories, and data were processed using Neolynx software (Neolynx Inc., CA, USA). Urine organic acids were analyzed by gas chromatography–mass spectrometry (GC–MS) on stored samples; this test provides a detailed biochemical profile that can be highly suggestive of organic acidemias in the appropriate clinical context. However, results were interpreted as screening-based presumptive findings unless confirmed by molecular and/or enzyme testing.

### Neuroimaging

All enrolled children underwent neuroimaging (CT and/or MRI) as part of the initial diagnostic work-up in the PICU after clinical stabilization. The timing and modality of neuroimaging were determined pragmatically based on the child's clinical condition, the urgency of the evaluation, and local imaging availability. Brain CT was typically used when rapid exclusion of acute structural pathology was required. In contrast, MRI was performed when clinically feasible to characterize suspected metabolic or non-structural brain injury better. MRI was acquired and interpreted according to the routine institutional neuroradiology workflow rather than a study-specific advanced research protocol.

### Outcomes and definitions

The primary outcome was the prevalence (yield) of abnormal metabolic screening (TMS and/or GC–MS) suggestive of an underlying IEM among children with presumed non-infectious encephalopathy (NIE) presented to the PICU. Secondary outcomes included (i) early clinical and biochemical predictors of abnormal metabolic screening at PICU admission and (ii) in-hospital mortality.

### Statistical analysis

Data were curated and analyzed using SPSS version 26 (SPSS Inc., Chicago, IL, USA). Normality was assessed using the Shapiro–Wilk test. Categorical variables were summarized as counts and percentages, whereas continuous variables were expressed as mean ± SD for approximately normally distributed data or median (IQR) for skewed data. Categorical variables were compared using the Chi-square test or Fisher's exact test, as appropriate. Continuous variables were compared between two groups using the independent-samples t-test for normally distributed variables and the Mann–Whitney U test for non-normally distributed variables. Comparisons across more than two groups were performed using one-way ANOVA for parametric data and the Kruskal–Wallis test for non-parametric data. Univariate binary logistic regression was used to identify admission factors associated with abnormal metabolic screening (TMS and/or GC–MS suggestive of an underlying IEM). Given the limited number of abnormal metabolic screening events, a parsimonious multivariable logistic regression model was then fitted using the strongest clinically relevant early predictors to reduce overfitting. A separate univariate logistic regression analysis was retained for in-hospital mortality as a secondary outcome. Odds ratios (ORs) are reported with 95% confidence intervals (95% CIs). Receiver operating characteristic (ROC) curve analysis was used to assess the discriminative performance of selected biomarkers, and optimal cut-off values were determined using the Youden index. Area under the ROC curve (AUC) values are reported with 95% confidence intervals (95% CIs). A two-tailed p-value ≤ 0.05 was considered statistically significant.

## Result

### Demographic and clinical characteristics

Between March 2023 and June 2024, 100 children with non-infectious acute encephalopathy (NIE) were recruited. The median age of the studied group was 8 months (IQR: 4–18 months), and 53% were females. As shown in Table 1, consanguinity was observed in 40% of cases, while a positive family history of similar illness or unexplained sibling death was reported in 21%. Regarding neurological manifestations, disturbed consciousness was the most frequent presentation (90%), followed by hypertonia (43%), delayed motor development (24%), delayed speech (22%), and cognitive delay (17%). Microcephaly and hypotonia were less common, observed in 5% and 4% of patients, respectively (Table 1). Seizures occurred in 60% of patients and were classified according to predominant clinical semiology as generalized-onset motor seizures in 46%, focal-onset seizures in 9%, myoclonic seizures in 3%, and mixed/unclassified seizure presentations in 2% (Table 1).

**Table 1:** Demographic and clinical characteristics of the studied children (N = 100)

Variable	Description/Category	n (%) or Median (IQR)
Age (months)		8 (4–18)
Gender	Male/Female	47 (47%)/53 (53%)
Consanguinity	Present	40 (40%)
Positive family history		21 (21%)
Neurological/developmental signs	Disturbed consciousness	90 (90%)
	Cognitive delay	17 (17%)
	Microcephaly	5 (5%)
	Delayed motor development	24 (24%)
	Hypotonia/Hypertonia	4 (4%)/43 (43%)
	Delayed speech	22 (22%)
Seizure classification	None	40 (40%)
	Generalized-onset motor seizures	46 (46%)
	Focal-onset seizures	9 (9%)
	Myoclonic seizures	3 (3%)
	Mixed/unclassified seizure presentations	2 (2%)
Outcome	Survived/Died	41 (41%)/59 (59%)

**Note:** Categorical variables are presented as frequency (%); continuous variables as median (IQR). Seizure classification was based on the predominant documented clinical seizure semiology at presentation

Overall, 59% of patients died, while 41% survived and were discharged from the pediatric intensive care unit (PICU).

### Laboratory findings

**Fig. 1:**
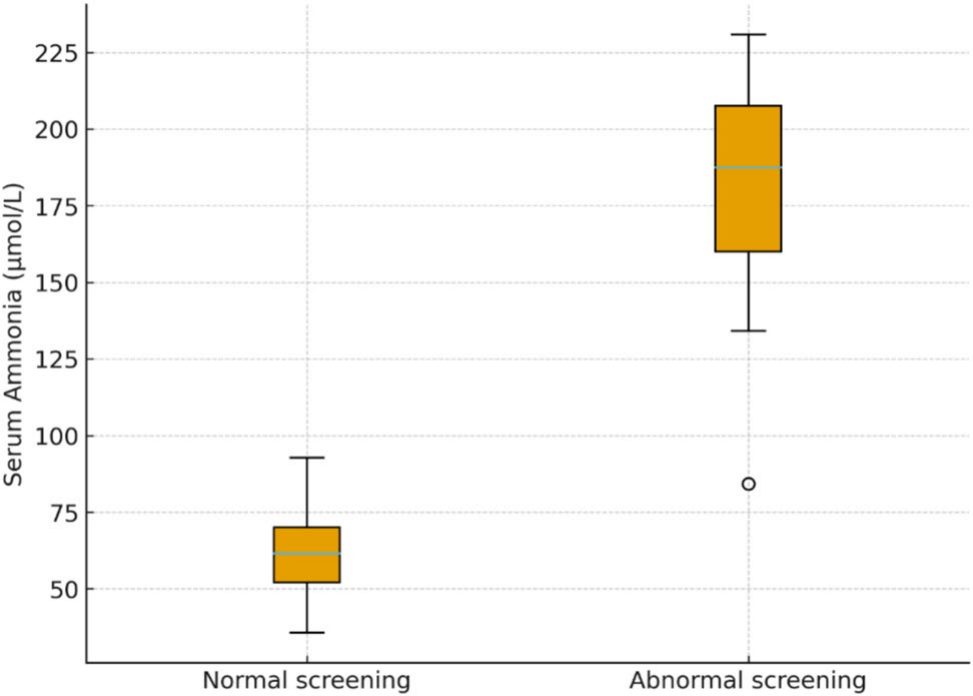
Distribution of serum ammonia levels among children with normal and abnormal metabolic screening. (The figure shows significantly higher ammonia values in the abnormal-screening group, p < 0.001.)

Baseline laboratory results of the studied cohort are presented in Table 2. The median serum ammonia level was 89 µmol/L (IQR: 55–169), while the median blood lactate concentration was 14.7 mmol/L (IQR: 12–19.4). The median serum glucose was 85 mg/dL (IQR: 73–98), and the mean PCO₂ and bicarbonate (HCO₃) levels were 39.6 ± 10.8 mmHg and 22 mmol/L (IQR: 13–25.9), respectively. When comparing patients with abnormal metabolic screening to those with expected results, serum ammonia and lactate levels were markedly higher in the abnormal group (p < 0.001 for both). As illustrated in Fig. 1, elevated ammonia (> 93.5 µmol/L) was a strong predictor of an abnormal metabolic profile on screening.

**Table 2:** Laboratory findings among children with acute non-infectious encephalopathy (N = 100)

Laboratory Parameter	Median (IQR) or Mean ± SD	Normal Range	Interpretation
Ammonia (µmol/L)	89 (55–169)	15–45	Elevated in 42% of patients
Lactate (mmol/L)	14.7 (12–19.4)	0.5–2.2	Elevated by 58%
Glucose (mg/dL)	85 (73–98)	70–110	Within normal limits
pH	7.30 (7.20–7.36)	7.35–7.45	Mild metabolic acidosis in 33%
PCO₂ (mmHg)	39.6 ± 10.8	35–45	Within normal range
HCO₃ (mmol/L)	22 (13–25.9)	22–28	Reduced in 29% of cases

Data expressed as median (IQR) or mean ± SD, as appropriate

### Neuroimaging findings

All studied children underwent neuroimaging (CT and/or MRI) for the diagnostic evaluation. Abnormal neuroimaging findings were detected in 64% of patients, while 36% had regular scans. The most frequent abnormalities were diffuse brain edema (26%), subcortical white matter hypodensities (22%), and basal ganglia lesions (10%). Less frequent findings included cerebral atrophy (4%) and focal cortical hypodensities (2%). No cases showed intracranial hemorrhage or mass effect. The distribution of neuroimaging abnormalities is summarized in Table 3.

**Table 3:** Neuroimaging findings among children with acute non-infectious encephalopathy (N = 100)

Neuroimaging Feature	n (%)	Description/Interpretation
Normal imaging	36 (36%)	No pathological changes detected
Diffuse brain edema	26 (26%)	Generalized cerebral swelling with effacement of sulci and ventricles
Subcortical white matter hypodensities	22 (22%)	Bilateral patchy low-attenuation lesions suggestive of metabolic insult
Basal ganglia lesions	10 (10%)	Relative distribution of metabolic disorders identified
Cerebral atrophy	4 (4%)	Global cortical thinning and ventricular dilatation
Focal cortical hypodensities	2 (2%)	Localized low-density regions, possibly ischemic
Total abnormal imaging	64 (64%)	

Data are presented as numbers (%)

### Metabolic screening results (TMS & GC–MS)

Metabolic screening was performed for all 100 children using tandem mass spectrometry (TMS) for plasma acylcarnitines and amino acids, and gas chromatography–mass spectrometry (GC–MS) for urinary organic acids. Abnormal metabolic screening results were detected in 26 children (26%), suggestive of an underlying inborn error of metabolism (IEM). As summarised in Table 4, the most common metabolic abnormalities were amino acid disorders (38.5%), followed by organic acidemias (34.6%), and fatty acid oxidation defects (26.9%).

**Table 4:** Distribution of metabolic abnormalities detected by TMS and GC–MS (n = 26 abnormal cases)

Category	Most likely diagnoses suggested by the biochemical profile	n (%)
Amino acid disorders	Urea-cycle defects (6), MSUD (4)	10 (38.5%)
Organic acidemias	Methylmalonic acidemia (5), Propionic acidemia (4)	9 (34.6%)
Fatty acid oxidation defects	MCAD (4), MADD (3)	7 (26.9%)
Total		26 (100%)

Data are presented as numbers and percentages of cases with abnormal metabolic screening. Note: Listed diagnoses reflect biochemical profiles suggestive of each disorder and were not confirmed by molecular/genetic testing

Within the amino acid disorders, urea cycle defects and maple syrup urine disease (MSUD) were the most frequent. Methylmalonic acidemia (MMA) and propionic acidemia (PA) predominate among organic acidemias. Fatty acid oxidation defects mainly include medium-chain acyl-CoA dehydrogenase deficiency (MCAD) and multiple acyl-CoA dehydrogenase deficiency (MADD). The proportional distribution of metabolic categories is summarized in Table 4.

### Predictors of abnormal metabolic screening

Univariate logistic regression for the primary diagnostic outcome showed that higher serum ammonia, cognitive delay, and delayed motor development were associated with abnormal metabolic screening suggestive of an underlying IEM. Specifically, serum ammonia was associated with abnormal screening in univariate analysis (OR 1.009 per 1 µmol/L increase, 95% CI 1.004–1.015; p < 0.001), as were cognitive delay (OR 4.37, 95% CI 1.47–13.01; p = 0.008) and delayed motor development (OR 3.44, 95% CI 1.29–9.18; p = 0.014). Given the limited number of abnormal screening events, a parsimonious multivariable logistic regression model was fitted. In this model, higher serum ammonia (adjusted OR 1.010, 95% CI 1.004–1.015; p = 0.001) and delayed motor development (adjusted OR 3.66, 95% CI 1.24–10.86; p = 0.019) remained independent predictors of abnormal metabolic screening (Table 5).

**Table 5:** Univariate and parsimonious multivariable logistic regression for predictors of abnormal metabolic screening suggestive of an underlying IEM (N = 100)

Predictor	Unadjusted OR (95% CI)	p-value	Adjusted OR (95% CI)	p-value
Serum ammonia (per 1 µmol/L increase)	1.009 (1.004–1.015)	< 0.001	1.010 (1.004–1.015)	0.001
Cognitive delay	4.37 (1.47–13.01)	0.008	—	—
Delayed motor development	3.44 (1.29–9.18)	0.014	3.66 (1.24–10.86)	0.019

Abnormal metabolic screening was defined as TMS and/or GC–MS findings suggestive of an underlying IEM. The multivariable model was intentionally restricted to a parsimonious set of predictors because only 26 children had abnormal metabolic screening

### Comparison of radiological findings according to metabolic screening results

Children with abnormal metabolic screening showed a higher frequency of positive neuroimaging findings, particularly brain atrophy, than those with normal screening results. CT data were available for all 100 children, whereas MRI was performed in 84 children. However, as shown in Table 6, these differences did not reach statistical significance (p > 0.05).

**Table 6:** Comparison of CT and MRI findings according to metabolic screening results

Modality (denominator)	Radiological finding	Normal screening	Abnormal screening	p-value
CT (n = 100)	Normal	34/74 (45.9%)	12/26 (46.2%)	0.64
	Brain atrophy	28/74 (37.8%)	14/26 (53.8%)	
	Dural sinus thrombosis	1/74 (1.4%)	0/26 (0.0%)	
	Diffuse subcortical hypodensity	3/74 (4.1%)	0/26 (0.0%)	
	Hydrocephalus	2/74 (2.7%)	0/26 (0.0%)	
	Brain edema	2/74 (2.7%)	0/26 (0.0%)	
	Intracranial hemorrhage	2/74 (2.7%)	0/26 (0.0%)	
	Few calcified foci	2/74 (2.7%)	0/26 (0.0%)	
MRI (n = 84)	Negative or normal	49/62 (79.0%)	13/22 (59.1%)	0.18
	Positive (any)	13/62 (21.0%)	9/22 (40.9%)	
	Brain atrophy	12/62 (19.4%)	4/22 (18.2%)	

**Note:** CT was available for all enrolled children (74 with normal metabolic screening and 26 with abnormal metabolic screening). MRI was performed in 84 children (62 with normal metabolic screening and 22 with abnormal metabolic screening). Percentages are calculated within each modality-specific subgroup

### Clinical correlates by TMS category

As shown in Table 7, neurodevelopmental impairments differed across TMS-defined categories. Cognitive delay and delayed motor development were more frequent among children with protein and lipid metabolism defects, whereas hypotonia was more frequent in organic acidemias. Median ammonia levels were significantly higher in protein and lipid defects than in routine screening and organic acidemia groups (all p < 0.05).

**Table 7:** Clinical and biochemical correlations across TMS categories

Variable	Normal (n = 74)	Protein defects	Lipid defects	Organic acidemias	Carbohydrate/Urea-cycle
Cognitive delay	Lower	Higher*	Higher*	—	—
Delayed motor development	Lower	Higher*	Higher*	—	—
Hypotonia	Lower	—	—	Higher*	—
Ammonia (median)	Lower	Higher*	Higher*	Lower vs protein/lipid*	—

^*^p < 0.05 for between-group comparisons reported in the original text

Note: Exact counts by TMS subgroup were not provided; this table summarizes significant directions of effect as reported

### Univariate logistic regression for mortality

As a secondary outcome analysis, univariate binary logistic regression showed that higher serum ammonia, PCO₂, and HCO₃ were associated with increased odds of in-hospital mortality. The corresponding odds ratios, 95% confidence intervals, and p-values are presented in Table 8.

**Table 8:** Univariate logistic regression for in-hospital mortality (secondary outcome)

Predictor	Odds Ratio (OR)	95% CI	p-value
Ammonia (per 1 µmol/L increase)	1.007	1.002–1.013	0.012
PCO₂ (per 1 mmHg increase)	1.045	1.005–1.086	0.028
HCO₃ (per 1 mmol/L increase)	1.054	1.002–1.110	0.041

### ROC Analysis for diagnostic and prognostic performance

**Fig. 2:**
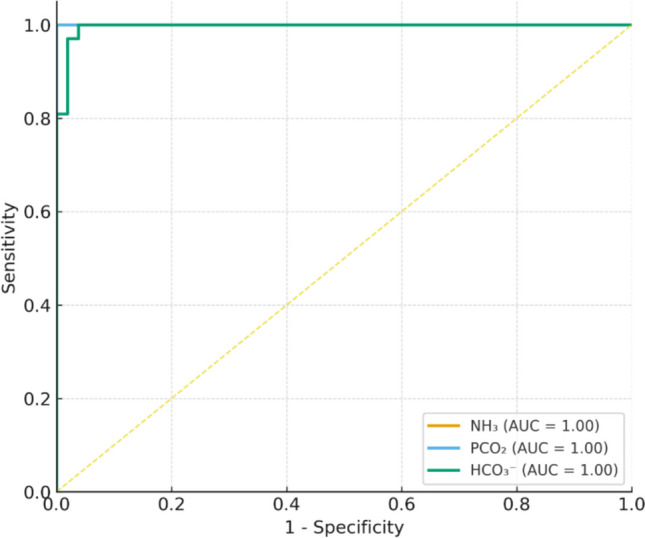
Receiver operating characteristic (ROC) curves for serum ammonia (NH₃), PCO₂, and HCO₃ to predict mortality among children with acute non-infectious encephalopathy. Curves illustrate the overall discriminative performance of each biomarker; AUC values are shown in the legend

Receiver operating characteristic (ROC) analysis was used to evaluate the diagnostic performance of serum ammonia for abnormal metabolic screening and the prognostic performance of serum ammonia, PCO₂, and HCO₃ for in-hospital mortality. Optimal cut-off values were selected using the Youden index. As shown in Table 9, serum ammonia demonstrated fair discrimination for abnormal metabolic screening (AUC 0.776, 95% CI 0.665–0.866) and modest prognostic discrimination for mortality (AUC 0.669, 95% CI 0.560–0.778). PCO₂ and HCO₃ also showed modest discriminative ability for mortality, with AUCs of 0.643 (95% CI 0.521–0.754) and 0.614 (95% CI 0.493–0.723), respectively. The corresponding ROC curves are shown in Fig. 2.

**Table 9:** ROC analysis for diagnostic and prognostic biomarkers

Outcome	Marker	Cut-off*	Sensitivity	Specificity	AUC (95% CI)
Abnormal metabolic screening	Ammonia	> 98.0 µmol/L	84.6%	64.9%	0.776 (0.665–0.866)
Mortality	Ammonia	> 61.0 µmol/L	81.4%	51.2%	0.669 (0.560–0.778)
Mortality	PCO₂	> 35.9 mmHg	78.0%	56.1%	0.643 (0.521–0.754)
Mortality	HCO₃	> 24.3 mmol/L	44.1%	78.0%	0.614 (0.493–0.723)

^*^ Optimal cut-off values were determined using the **Youden index**

## Discussion

Identifying and managing IEM disorders is crucial for mitigating morbidity and mortality for children presenting with acute non-infective encephalopathy [10]. This study characterised and estimated the IEMs clinical burden in a tertiary PICU setting after initial evaluation suggestive of no overt CNS infection. We found that 26% of children with unexplained acute encephalopathy had abnormal metabolic screening, which is suggestive of an underlying IEM. This proportion can be compared with that of the cohort from Egypt reported by Maksoud et al. (33%) [11] but is higher than that reported by Kirti et al. from India (7.2%) [7].

This discrepancy among studies highlights differences in an individual's genetic background, consanguinity, referral patterns, and the scope of local screening panels.

Dominating the clinical pictures of our cohort were the following: disturbed consciousness, seizures (predominantly generalized-onset motor seizures), hypertonia, and delays in attaining motor and speech milestones. Such patterns correspond with previously documented regional and international studies demonstrating that impaired neurodevelopment is a universal hallmark of IEMs [12–14]. Consanguinity and a positive family history—both widely prevalent in our series—reinforce the genetic predisposition and the importance of family counselling and cascade screening in the still understudied high-risk populations [13, 14].

More children with abnormal metabolic screenings had intellectual disability, delayed motor development, and were more likely to present with hypotonia than children with metabolic screens that yielded expected results. Among the metabolic subtypes, the strongest association with neurodevelopmental impairment was observed for abnormal protein and lipid metabolism. Conversely, the strongest correlations with hypotonia were observed in patients with organic acidemias, a finding consistent with prior clinical observations [15]. Among the children with protein and lipid metabolism defects, higher-than-normal median ammonia levels were observed and are consistent with the pathophysiology of urea-cycle and amino-acid catabolic disturbances [15].

At PICU admission, higher serum ammonia and delayed motor development were the most informative predictors of abnormal metabolic screening suggestive of an underlying IEM. In logistic regression, serum ammonia was associated with abnormal metabolic screening both in univariate analysis (OR 1.009 per 1 µmol/L increase, 95% CI 1.004–1.015; p < 0.001) and after adjustment for delayed motor development (adjusted OR 1.010, 95% CI 1.004–1.015; p = 0.001). Delayed motor development also remained independently associated with abnormal metabolic screening (adjusted OR 3.66, 95% CI 1.24–10.86; p = 0.019). These findings reinforce the value of combining a rapidly available biochemical marker with developmental history when prioritizing urgent metabolic work-up in children with presumed non-infectious encephalopathy.

These findings demonstrate the clinical utility of widely accessible prognostic markers for the early assessment of encephalopathy in children, reaffirming the need to simplify laboratory marker selection (ammonia, lactate, glucose, acid–base status) in cases of suspected IEM “[6]”.

The most prevalent neuroimaging anomalies among our cohort—subtle diffuse cerebral oedema, augmented subcortical white matter, and, less frequently, basal ganglia lesions—contrasted with the classical features of metabolic encephalopathy [16]. No single CT/MRI feature reached statistical significance, yet the discrepancy between the screened normal and abnormal groups, as a whole, is uninformative; the repetitive patterns are pathophysiologically tenable and mechanically corroborative, given the biochemistry [16].

Regarding distribution, amino acid disorder profiles were most frequent, followed by organic acidemia profiles and fatty acid oxidation defect profiles (Table 4). Differences across studies may reflect genetic background, consanguinity, referral patterns, and the scope of available screening panels [14, 17].

The recorded 41% mortality rate speaks to the metabolic burden of encephalopathy in critically ill children and the overwhelming need to recognise and address it within a defined approach. In turn, these results strengthen the clinical rationale for conducting additional metabolic screens in children with unexplained encephalopathy, as well as for initiating ammonia-lowering interventions early in the care pathway upon diagnosis of hyperammonemia.

The forensic consequences of this are significant. The postmortem examination may show elevated ammonia, combined with a disturbed acid–base balance that may be misinterpreted as evidence of toxic or hypoxic encephalopathy. The systematic application of metabolic screening within forensic pediatric autopsy evaluations investigating deaths of unknown cause has the potential to better delineate the presence of neglected inborn metabolic disorders, preventing their misclassification as unnatural deaths.

## Forensic perspective

The metabolic alterations identified in this cohort have diagnostic and forensic relevance. An example would be during postmortem examinations in unexplained pediatric deaths. Biochemical patterns that may raise suspicion of an underlying metabolic disorder (e.g., hyperammonemia and organic acid patterns) can be informative but are not disease-specific and should be interpreted in the clinical context.

## Strengths and limitations

The advantages include systematic phenotyping, TMS, GC–MS, and the integration of clinical, biochemical, and radiological data. Limitations include the single-centre design, a small sample size, and the absence of validating molecular tests that would have clarified subtype classification and genotype–phenotype correlations. Future multicenter studies incorporating genomic sequencing are needed to describe the molecular landscape of IEMs in the Egyptian and regional pediatric population and to develop cost-effective screening strategies. Additionally, microbiological cultures and/or molecular infectious testing were not obtained systematically for all enrolled children, as these investigations were performed selectively based on clinical judgment and local availability rather than as mandatory protocol-based tests. Therefore, some cases may have included unrecognized infectious contributors despite initial classification as presumed non-infectious encephalopathy. Likewise, comprehensive toxicology screening was not systematically available, so occult toxic exposures cannot be entirely excluded.

## Implications

In lower-resourced situations, intertwining routine metabolic evaluations with ammonia and lactate assays could accelerate diagnosis and lifesaving treatment. This data supports greater standardisation of PICU hyperammonemia protocols, earlier referrals to metabolic specialists, and family counselling programs that align with IEM clinical pathways and best-practice overviews [6, 10, 18].

IEMs remain a significant portion of NIE in children in our region. Attaching biochemical symptoms [especially ammonia; see above], targeted metabolic assessment TMS/GC–MS, and adjunctive neuroimaging improves diagnostic and survival yield, albeit with molecular diagnostics more obtainable for targeted and preventative care [13–18].

## Conclusion

In the tertiary PICU setting, abnormal metabolic screening suggestive of an underlying IEM was identified in 26% of children with presumed non-infectious encephalopathy, with an overall mortality of59 %. Of the metabolic disorders, the most prevalent amino-acid and organic-acid abnormalities, combined with hyperammonemia and lactic acidosis, underscore concerning metabolic disturbances. Simple early markers, particularly serum ammonia and delayed motor development, may help prioritize children with presumed non-infectious encephalopathy for urgent metabolic work-up, targeted treatment, and confirmatory testing.

Neuroimaging findings of metabolic imaging (diffuse oedema with subcortical white-matter structures) were supported—though not confirmed—by the biochemical workup. These cases forcefully argue for early routine metabolic screening with TMS/GC-MS for unexplained encephalopathy and overt protocolized hyperammonemia, and for integrating metabolic medicine into the PICU team. To tailor epidemiology and enhance precision care, multicenter investigations with molecular confirmation of diagnosis should be conducted, and organized methodological approaches should be developed for high-risk groups, including scalable screening and family support. Our findings may also assist clinicians investigating the sudden and unexplained demise of children and provide insights into the consideration of metabolic encephalopathy as a contributing factor [19].

## Data Availability

Data will be made available on request.

## References

[1] Hon KLE, Leung KKY, Kwok AM-K, Belaramani KM (2021) Inborn errors of metabolism in critically ill children: initial acute care guide. Pediatr Emerg Care. 10.1097/PEC.0000000000002479 34180862 10.1097/PEC.0000000000002479

[2] Fukao T, Nakamura K (2019) Advances in inborn errors of metabolism. J Hum Genet 64:65 30679804 10.1038/s10038-018-0535-7

[3] Stenton SL, Kremer LS, Kopajtich R et al (2020) The diagnosis of inborn errors of metabolism by an integrative “multi-omics” approach: a perspective encompassing genomics. J Inherit Metab Dis. 43:25–35 31119744 10.1002/jimd.12130

[4] El-Hattab AW, Almannai M, Sutton VR (2018) Newborn screening: history, current status, and future directions. Pediatr Clin North Am 65:389–405 29502920 10.1016/j.pcl.2017.11.013

[5] Yang C, Zhou C, Xu P et al (2020) Newborn screening and diagnosis of inborn errors of metabolism: a 5-year study in an eastern Chinese population. Clin Chim Acta 502:133–138 31893530 10.1016/j.cca.2019.12.022

[6] Saudubray JM, Garcia-Cazorla À (2018) Inborn errors of metabolism: overview of pathophysiology, manifestations, evaluation, and management. Pediatr Clin North Am 65:179–208 29502909 10.1016/j.pcl.2017.11.002

[7] Singhal K, Bothra M, Kapoor S, Jhamb U, Mishra D (2022) Metabolic disorders among children with acute encephalopathy. Indian J Pediatr 89(7):665–672 35254636 10.1007/s12098-022-04087-2

[8] Yang CJ, Wei N, Li M et al (2018) Diagnosis and therapeutic monitoring of inborn errors of metabolism in 100,077 newborns from Jining City in China. BMC Pediatr 18:110 29534692 10.1186/s12887-018-1090-2 PMC5850921

[9] Lampret BR, Murko S, Tansek M et al (2015) Selective screening for metabolic disorders in the Slovenian pediatric population. J Med Biochem 34:58–63 28356825 10.2478/jomb-2014-0056 PMC4922335

[10] Huang X, Yang L, Tong F et al (2012) Screening for inborn errors of metabolism in high-risk children: a 3-year pilot study in Zhejiang Province. China BMC Pediatr 12:18 22364411 10.1186/1471-2431-12-18 PMC3306752

[11] Maksoud MA, El-Sayed SM, Shatla RH et al (2018) Frequency of inborn errors of metabolism in children with unexplained acute encephalopathy at an emergency department. Neuropsychiatr Dis Treat 14:1715–1720 29988750 10.2147/NDT.S165833 PMC6029674

[12] Shawky RM, Abd-Elkhalek HS, Elakhdar SE (2015) Selective screening in neonates suspected to have inborn errors of metabolism. Egypt J Med Hum Genet 16:165–171

[13] Khalaf SM, El-Tellawy MM, Refat NH et al (2019) Detection of some metabolic disorders in suspected neonates admitted to Assiut University Children’s Hospital. Egypt J Med Hum Genet 20:29

[14] Selim LA, Hassan SA, Salem F et al (2014) Selective screening for inborn errors of metabolism by tandem mass spectrometry in Egyptian children: a 5-year report. Clin Biochem 47:823–828 24731791 10.1016/j.clinbiochem.2014.04.002

[15] Jayashree KR, Madhivanan S, Kumarasamy K, Karthick AR (2020) Clinical spectrum of inborn errors of metabolism in children in a tertiary care hospital. Int J Contemp Pediatr. 10.18203/2349-3291.ijcp20200500

[16] Palculict ME, Zhang VW, Wong LJ, Wang J (2016) Comprehensive mitochondrial genome analysis by massively parallel sequencing. Methods Mol Biol 1351:3–17 26530670 10.1007/978-1-4939-3040-1_1

[17] Cheema HA, Malik HS, Parkash A, Fayyaz Z (2016) Spectrum of inherited metabolic disorders in Pakistani children presenting at a tertiary care centre. J Coll Physicians Surg Pak 26(6):498–502 27353988

[18] Fletcher JM (2016) Metabolic emergencies and the emergency physician. J Paediatr Child Health 52:227–230 27062628 10.1111/jpc.13106

[19] Sharma S, Prasad AN (2017) Inborn errors of metabolism and epilepsy: current understanding, diagnosis, and treatment approaches. Int J Mol Sci 18(7):1384 28671587 10.3390/ijms18071384 PMC5535877

